# Through Patients' Eyes: Evaluating the Consent Process in Elective Orthopedic Surgery at a Tertiary Referral Center in Ireland

**DOI:** 10.7759/cureus.61801

**Published:** 2024-06-06

**Authors:** Abobaker Younis, Mehad Elmubarak, Hussam Elkhwad

**Affiliations:** 1 Orthopaedics and Traumatology, University Hospital Galway, Galway, IRL

**Keywords:** patient satisfaction, patients’ perspective, ireland, galway, informed consent

## Abstract

Background: Informed consent is a critical component of ethical clinical practice, particularly in elective orthopedic procedures. It ensures that patients understand the nature, benefits, and risks of the medical procedures they agree to undergo. This study aims to evaluate the informed consent process at Merlin Park University Hospital from the patient's perspective.

Methods: This cross-sectional observational study included 140 participants undergoing elective orthopedic procedures. Data were collected via a questionnaire focusing on socio-demographic information and the informed consent process, including details on who provided the information, where and when the consent was signed, and patient understanding and satisfaction. Responses were analyzed using SPSS version 26 (IBM Corp., Armonk, NY).

Results: The majority of participants were females, with 80 (57.1%) women and 60 (42.9%) men. The average age of the participants was 60.86 years. Most participants were employed, constituting 75 (53.6%) of the sample, and 55 (39.3%) had attained university or higher education. The most common procedures were total hip replacement, with 30 (21.4%) participants, and total knee replacement, with 20 (14.3%) participants. Information during the consent process was primarily provided by consultants in 80 (57.1%) cases. High satisfaction levels were reported, with 139 (99.3%) participants expressing satisfaction. Significant correlations were found between satisfaction and the type of healthcare provider, type of operation, and educational level.

Conclusion: The informed consent process at Merlin Park University Hospital is generally effective, with high patient satisfaction and understanding. However, there is a preference for concise information delivery. Enhancing the process through personalized information delivery could further improve patient satisfaction and comprehension. These findings contribute valuable insights into patient-centered care and informed consent in elective orthopedic surgeries.

## Introduction

Informed consent is a cornerstone of ethical clinical practice, ensuring that patients are fully aware of the nature, benefits, and risks associated with medical procedures before agreeing to them. The principle of informed consent is rooted in the respect for patient autonomy, emphasizing the patient's right to make decisions about their own health care based on comprehensive and understandable information. This process is especially critical in elective orthopedic procedures, where patients often face a range of surgical options and potential outcomes [1].

Despite its crucial role, the effectiveness of the informed consent process is frequently judged by the comprehensiveness of the consent forms rather than by patient understanding and engagement. Research has shown that a significant proportion of patients do not fully understand the information provided during the consent process, which raises concerns about the true nature of their consent [2,3]. These studies suggest a disconnect between the information delivered and the patient's comprehension, indicating a gap in the informed consent process that could impact patient outcomes.

The majority of existing studies have focused on the content and clarity of consent forms themselves, often neglecting how patients perceive and internalize this information [4]. This oversight underscores the necessity of reassessing our approach to informed consent, emphasizing the patient's perspective to ensure that they are genuinely informed and comfortable with the decisions they make regarding their health care.

The consent process typically begins during the initial visit to the clinic, where the primary details of potential surgical interventions are discussed. It extends through interactions with various healthcare providers, including arthroplasty nurses and staff at pre-assessment clinics, and culminates with the final signature obtained typically on the morning of surgery. Each of these steps represents a crucial opportunity for reinforcing patient understanding and ensuring all questions are adequately addressed.

Given this backdrop, the current audit aims to investigate the informed consent process at Merlin Park University Hospital (MPUH) from the patient's perspective, assessing not just the informational content but also how effectively this information is communicated and understood. By focusing on patient comprehension and recall at each stage of the consent process, this project seeks to identify key areas for improvement, thereby enhancing patient satisfaction and outcomes in elective orthopedic procedures.

In contrast to the study by Davey et al. (2021), which focused on the quality of informed consent documentation for orthopedic trauma patients in an emergency setting, the current study delves into the informed consent process from the patients' perspective, assessing their understanding and satisfaction with the information provided [5]. This shift from a procedural focus to a patient-centered approach offers a more holistic view of the effectiveness of informed consent in orthopedic surgeries [6].

## Materials and methods

This study was designed as a cross-sectional observational audit to assess patient understanding and recall of the informed consent process at MPUH The focus was on elective orthopedic procedures, particularly exploring the patient perspective in understanding the components of informed consent, which includes information disclosure, competency, voluntariness, comprehension, and consent itself.

Study setting and period

The study was conducted at MPUH, a busy tertiary referral center renowned for its extensive range of elective orthopedic services. The elective orthopedic surgery department conducts five to eight consultant-led elective lists weekly, accommodating a broad spectrum of orthopedic interventions. The procedures include but are not limited to total hip and knee replacements, arthroscopic surgeries for sports and shoulder injuries, upper limb surgeries, foot and ankle surgeries, and complex spinal surgeries. This diverse surgical activity reflects the hospital's comprehensive approach to orthopedic care, making it an ideal setting for examining the informed consent process across various types of procedures. The study was carried out over a two-month period from March to April 2023.

Participants

Participants included all post-surgical patients over the age of 16 years who were admitted to the orthopedic ward and agreed to participate in the study. Those requiring special care or intensive care, as well as patients admitted for symptomatic injections, were excluded from the study.

Data collection and analysis

Data were collected using a direct questionnaire method. The questionnaire was divided into two parts. The first part gathered socio-demographic data, including information on age, gender, marital status, residency, and occupation. The second part focused on the informed consent process, containing questions related to who signed the consent, where it was signed, who provided the information, and where this took place. It also sought information on the nature of the surgery, indications, possible complications, alternatives to surgery, expected hospital stay, complications if surgery is not performed, expected benefits, type of anesthesia used, and whether patients had the opportunity to ask questions. Responses from the questionnaire were analyzed using SPSS version 26 software (IBM Corp., Armonk, NY). The analysis focused on identifying patterns and discrepancies in patient recall and understanding of the informed consent process.

Ethical considerations

To protect patient confidentiality and ensure the ethical handling of data, patient identities were anonymized. Patients were informed that the data collected would only be used for scientific research purposes.

## Results

Demographic and basic information

The study included 140 participants, characterized by a slight female majority at 80 (57.1%) and males at 60 (42.9%). The age range of participants spanned from 34 to 80 years, with an average age of 60.86 (SD ± 11.25), reflecting a broad spectrum relevant to orthopedic care. Most participants were employed, constituting 75 (53.6%) of the sample, and 55 (39.3%) had attained university or higher education, as shown in Table 1.

**Table 1:** Demographic characteristics of the participants. This table summarizes the demographic characteristics of the 140 study participants. It includes the distribution of gender, mean age along with standard deviation, employment status, and education level.

Demographic variable	Frequency	Percentage (%)
Gender		
- Male	60	42.9
- Female	80	57.1
Age		
- Mean (SD)	60.86 (11.25)	
Employment status		
- Employed	75	53.6
- Unemployed	65	46.4
Education level		
- University/higher	55	39.3
- Below university	85	60.7

Types of operations and decision to proceed with surgery

Total knee replacement and total hip replacement were the most common procedures at 20 (14.3%) and 30 (21.4%), respectively, reflecting common orthopedic needs. A significant majority of patients, 95 (67.9%), made surgery decisions independently (without influence from family or friends), highlighting the importance of self-decision in elective surgeries, as shown in Table 2.

**Table 2:** Types of elective orthopedic procedures done during the study period. This table presents the different types of elective orthopedic procedures performed on the study participants. The frequency and percentage of each type of surgery, including total knee replacement, total hip replacement, carpal tunnel release, rotator cuff repair, and other procedures, are shown.

Type of operation	Frequency	Percentage (%)
Total knee replacement	20	14.3
Total hip replacement	30	21.4
Carpal tunnel release	10	7.1
Rotator cuff repair	15	10.7
Other	65	46.4

Informed consent process

Information during the consent process was typically provided by a combination of consultants, registrars, and senior house officers, supporting collaborative patient education, as shown in Table 3. The majority of consent forms, 115 (82.1%), were signed in the ward, suggesting that many decisions are finalized post admission.

**Table 3:** Details of source of information in the consent process. This table details the sources of information provided during the informed consent process. It includes the frequency and percentage of information provided by consultants, registrars, senior house officers, and others (nurses, interns, etc.).

Information provider	Frequency	Percentage %
Consultant	80	57.1%
Registrar	75	53.6%
Senior house officer	70	50%
Other	10	0.7%

**Figure 1:**
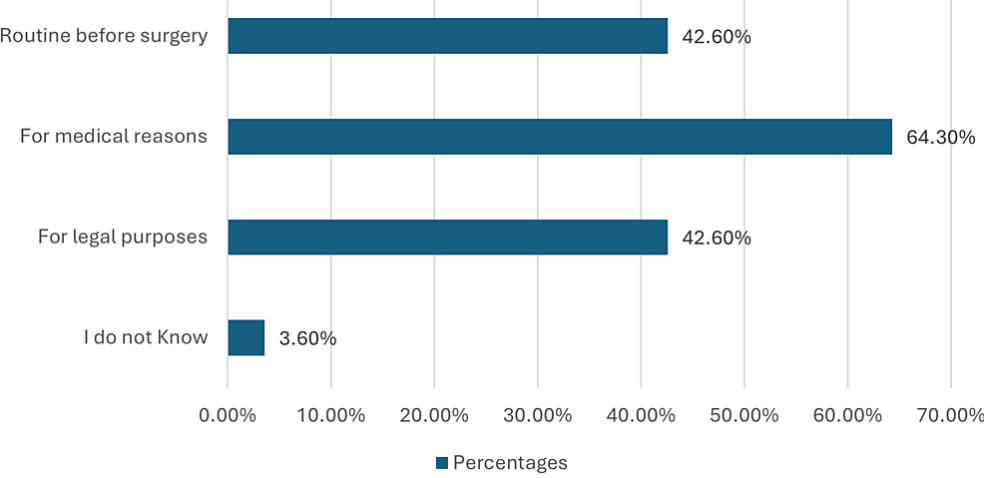
Importance of informed consent from patients’ perspective. This figure illustrates patients' perceptions of the importance of informed consent. It categorizes responses into reasons such as routine before surgery, medical reasons, legal purposes, and lack of knowledge (N ≠ 100%).

Patients' perceptions of the importance of informed consent are illustrated in Figure 1, categorizing responses into reasons such as routine before surgery, medical reasons, legal purposes, and lack of knowledge.

Patients' perception of consent and overall satisfaction

**Figure 2:**
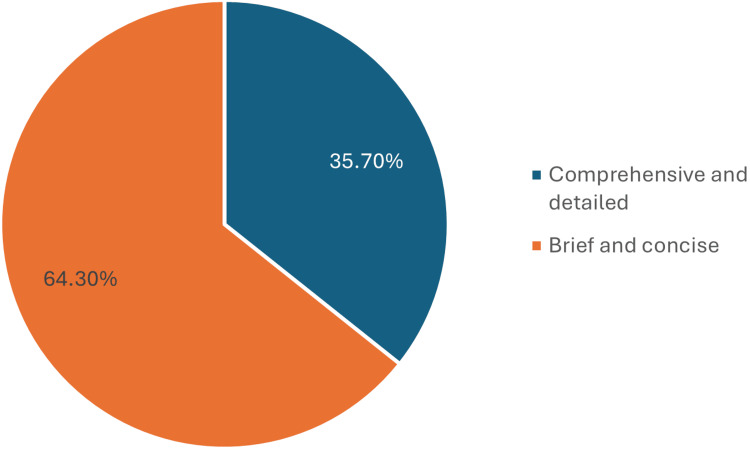
Patients preference for information in the consent process. This figure represents the patients' preferences for the type of information detail provided during the informed consent process. It shows the proportion of participants who preferred comprehensive and detailed information versus those who preferred brief and concise information.

A significant portion of the cohort, 90 (64.3%), preferred concise and brief information over a detailed comprehension approach, as seen in Figure 2.

**Figure 3:**
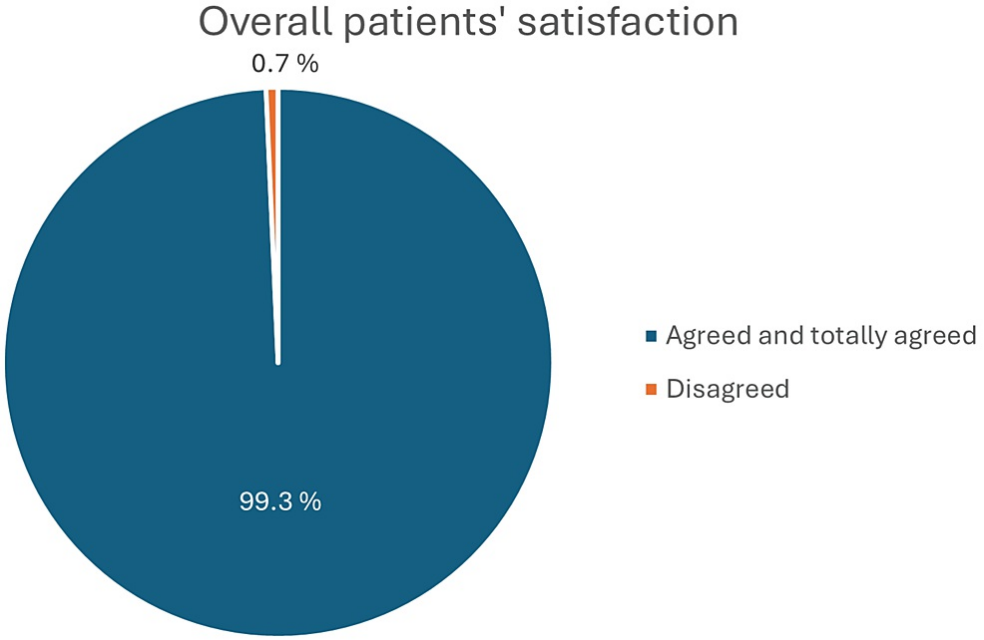
Overall patient satisfaction. This figure displays the overall satisfaction levels of patients with the informed consent process. It shows the percentage of participants who agreed or totally agreed that they were satisfied, as well as those who disagreed.

High satisfaction levels were noted, with 139 (99.3%) participants expressing satisfaction, indicating effective communication during the consent process, as displayed in Figure 3.

Specific elements of consent

All participants were informed about the necessity for surgery, and detailed surgical procedures were communicated to every participant. Nearly all, 135 (96.4%), understood the expected benefits, and everyone was aware of potential risks. Most participants were also informed about the expected hospital stay, 130 (92.9%), and alternatives to surgery, 95 (67.9%), as summarized in Table 4.

**Table 4:** Patients recalling informed consent elements that were discussed with them. This table summarizes the elements of the informed consent that patients recalled being discussed with them. It includes the frequency and percentage of patients who remembered discussions about the necessity for surgery, detailed procedures, expected benefits, potential risks, expected hospital stay, alternatives to surgery, and the opportunity to ask questions.

Informed consent element	Frequency	Percentage (%)
Necessity for surgery	140	100%
Detailed procedures	140	100%
Expected benefits	135	96.4%
Potential risks	140	100%
Expected hospital stay	130	92.9%
Alternatives to surgery	95	67.9%
Opportunity to ask questions	140	100%

Correlations and statistical significance

The data revealed a significant correlation between the type of healthcare provider and patient satisfaction levels. Patients who received information from consultants, either alone or in combination with other healthcare providers, showed a higher satisfaction rate, with 80 (57.14%) patients reporting having a consultant involved in their informed consent process. Patient satisfaction also varied significantly with the type of operation. Total hip replacement and total knee replacement reported higher satisfaction ratings. Additionally, the analysis indicated that educational level significantly influences satisfaction, with patients possessing university or higher education displaying higher satisfaction rates. Statistical tests confirmed that gender, operation type, and provider type had significant impacts on patient satisfaction, with all Pearson chi-square test results indicating strong associations between these factors, as shown in Table 5.

**Table 5:** Correlations and statistical significance. This table presents the results of statistical tests examining the correlation between various demographic and procedural variables and patient satisfaction levels. It includes the chi-square value, p-value, and whether the correlation is statistically significant for each variable.

Variable	Chi-square value	P-value	Significant (Yes/No)
Gender and satisfaction	19.605	<0.001	Yes
Operation type and satisfaction	89.995	<0.001	Yes
Provider type and satisfaction	73.951	<0.001	Yes
Education level and satisfaction	23.092	<0.001	Yes

## Discussion

The present study evaluated the informed consent process for elective orthopedic surgeries at MPUH, focusing on patient perspectives concerning the comprehensiveness and clarity of information provided. The study encompassed a broad age range and showed a significant female majority. Key findings include a strong preference for concise information, with high overall satisfaction regarding the consent process. Nearly all participants understood the necessity and details of their surgeries, with many aware of the associated risks and expected outcomes.

The high level of satisfaction and preference for concise information underscores the effectiveness of the current consent process but also highlights a potential area for improvement in tailoring information delivery to match patient preferences. The clinical implications are profound as ensuring patients are adequately informed can significantly impact their recovery and satisfaction, potentially reducing postoperative complications and legal claims associated with miscommunication.

Comparatively, studies from various parts of the world indicate variable patient understanding of informed consent content. For instance, a study conducted in the United States suggested that while the majority of patients felt informed, only a minority could recall specific details about risks and alternatives post-consent [7]. Similarly, research from the UK indicated improvements in patient satisfaction when additional visual aids were used during the consent process [8]. Additionally, a study by Sahin et al. in Turkey reported that while most patients felt informed about their surgery, only about half could recall specific surgical details several days post-consent [9]. These findings align with our observations at MPUH, suggesting that while satisfaction is high, comprehension of consent details could be enhanced through visual aids and reinforced information delivery over time.

At MPUH, all patients reported being informed about potential risks, contrasting with a US study where only 65% of orthopedic surgery patients could recall discussing risks at follow-up appointments [10]. Similarly, a Canadian study noted that only 80% of patients expressed satisfaction with the consent process compared to MPUH's 99.3% satisfaction rate, indicating the efficacy of MPUH's procedures in ensuring patient contentment [11]. These findings are further supported by research from Italy, where multimedia tools significantly improved patient understanding and satisfaction in informed consent processes [12], and from France, where a combination of verbal and written information improved overall understanding and satisfaction [13]. These studies suggest that MPUH's comprehensive risk communication is highly effective and could be further enhanced with multimedia tools and combined information delivery methods.

A strong preference for concise and clear information was noted at MPUH, whereas studies in Germany found that patients preferred more detailed explanations, with longer consultations leading to higher satisfaction [14]. This suggests that preferences for information detail can vary culturally and contextually. Moreover, two-thirds of patients at MPUH recalled discussing alternatives to surgery, compared to only 40% in a Japanese study, highlighting MPUH's superior performance in this crucial aspect of informed decision-making [15]. Research from Sweden also indicated that patients with higher education levels had a more comprehensive understanding of the informed consent contents [16], aligning with MPUH's findings that educational background significantly influences patient engagement and comprehension. Furthermore, studies from China and Brazil reported that digital consent forms and interactive multimedia presentations significantly improved patient understanding and recall, suggesting potential areas for MPUH to explore for further enhancing the consent process [17,18].

The study is not without limitations. The use of self-report measures for satisfaction and understanding may introduce response biases. Additionally, the single-center design may not fully capture the diversity of patient experiences across different regions or healthcare settings. Future research could explore multi-center studies to validate these findings and implement interventions like digital consent forms or multimedia presentations to enhance understanding across different patient demographics.

This project introduces novel insights into the patient-centered evaluation of the informed consent process in an Irish healthcare context, focusing on patient recall and satisfaction. Future studies could explore the impact of personalized consent processes tailored to individual patient needs and learning styles, potentially using adaptive digital platforms.

## Conclusions

This study reaffirms the importance of an effective informed consent process as crucial for patient autonomy and satisfaction. While patients at Merlin Park University Hospital generally reported high satisfaction, the study highlights opportunities for enhancing the consent process through more customized information delivery. These findings contribute valuable insights to the ongoing discourse on patient-centered care and informed consent in elective orthopedic surgeries.
